# Structural racism and intimate partner violence perpetration among racially diverse men transitioning into fatherhood: an anti-racist approach to IPV prevention

**DOI:** 10.1186/s40621-025-00562-4

**Published:** 2025-02-26

**Authors:** Tiara C. Willie, Sabriya Linton, Leslie B. Adams, Nicole M. Overstreet, Shannon Whittaker, Theresa Faller, Deja Knight, Trace S. Kershaw

**Affiliations:** 1 Department of Mental Health, Johns Hopkins Bloomberg School of Public Health, Baltimore, MD USA; 2 Department of Public Mental Health and Population Sciences, Stanford School of Medicine, Stanford, CA USA; 3 Department of Psychology, Clark University, Worcester, MA USA; 4 Center for Innovation in Social Science, Boston University, Boston, MA USA; 5 Department of Epidemiology, Johns Hopkins Bloomberg School of Public Health, Baltimore, MD USA; 6 Department of Social and Behavioral Sciences, Yale University School of Public Health, New Haven, CT USA

## Abstract

**Background:**

Young couples transitioning into parenthood are at elevated risk of experiencing intimate partner violence (IPV), in part, due to the social and economic stressors associated with this critical time. Interpersonal racial discrimination is a known risk factor for male-to-female IPV perpetration, however few studies have examined this relationship among men transitioning to fatherhood. Similarly, structural racism acknowledges how inequitable systems reinforce racial discrimination; yet, few studies have investigated whether structural racism relates to the discrimination-IPV perpetration association. This study examined relationships among structural racism, racial discrimination, stress, and IPV perpetration among racially diverse men transitioning into fatherhood.

**Methods:**

Using data from the 2007–2011 American Community Survey, a structural racism was assessed using a latent variable with four indicators: racial residential segregation, education inequity, income inequity, and employment inequity. Individual-level prospective data were collected during 2007–2011 from 296 men in expectant couples recruited from obstetrics, and ultrasound clinics in Connecticut. Structural equation models were conducted to investigate longitudinal associations among structural racism, discrimination, stress, optimism and emotional IPV perpetration.

**Results:**

Compared to white men, Black men were more likely to experience structural racism (B = 0.95, *p* <.001). Structural racism was associated with more racial discrimination (B = 0.45, *p* <.05), more stress (B = 0.40, *p* <.05), and less optimism (B=-0.50, *p* <.001). Racial discrimination was associated with more stress (B = 0.23, *p* <.05) and marginally associated with a greater likelihood to perpetrate emotional violence against a female partner (B = 0.23, *p* =.06). Stress was associated with a greater likelihood to perpetrate emotional violence against a female partner (B = 0.31, *p* =.05). The indirect path from structural racism to IPV perpetration via racial discrimination and stress was marginally significant (B = 0.05, *p* =.07).

**Conclusions:**

This study provides evidence of the ways in which structural racism in housing, education, income, and employment can contribute to men’s use of aggression and violence against a female partner. Future intervention efforts to reduce emotional IPV perpetration could benefit from addressing structural racism.

## Introduction

Preventing intimate partner violence (IPV) among young pregnant and parenting couples is a critical public health priority given its associations with significant adverse health outcomes [1–5]. Young mothers report more IPV experiences than non-parenting women [4, 6, 7]. Transitioning to parenthood can comprise both difficult and joyful times [8–10], but the developmental changes that occurs during this transition can produce additional stress in the family system and result in relationship conflicts [8]. Without effective IPV prevention programs, mothers experiencing IPV are more likely to experience chronic stress [1], rapid repeat pregnancies [11], depression [12], and children exposed to parental IPV are at elevated risk for future adolescent dating violence [2, 3, 5, 13]. 

## Racial disparities in IPV during prenatal and postnatal periods

Nearly 41% of women have experienced some form of sexual, physical, or psychological abuse by an intimate partner in their lifetime [14]; yet, there are significant racial and ethnic disparities in IPV experiences among young pregnant and parenting women. To date, the lifetime prevalence of IPV among Non-Hispanic Black women is 53.6%, and this group also has some of the highest estimates for 12-month experiences of IPV [14]. Using hospital and emergency department data in California, a recent study found that the prevalence of pregnancy-associated violence victimization was highest among non-Hispanic Black women [15]. Also, the risk of pregnancy-associated intimate partner homicide is three-fold among Black women compared to white and Hispanic women [16]. 

### Violent consequences of structural racism in the black community

Addressing the ways in which broader social-structural context influences IPV perpetration among Black men is one way to inform future tailored IPV prevention programs. A meta-analysis found that the emotional IPV perpetration is one of the most common forms of IPV perpetration among Black men [17]. IPV prevention in the Black community has to reconcile with the confluence of structural factors that have contributed to the marginalization and disenfranchisement of Black Americans in the larger society [18, 19]. 

Traditional Eurocentric theories of IPV have ignored how social-structural context contributes to IPV [18], but structural racism can provide more context to how structural inequalities influence perpetration of IPV among Black men. Structural racism refers to the social forces, institutions, ideologies and processes that reinforce inequities among racial and ethnic groups [20–22]. Specifically, structural racism captures how inequitable systems reinforce discriminatory beliefs through housing, education, employment, and criminal justice [21]. A growing body of research has investigated how social-structural context can influence the perpetration of IPV among Black men [18, 19, 23]. Accordingly, this conceptual research postulates that systems of oppression and power (e.g., racism, classism) can increase the risk of IPV perpetration. The compounding stress and frustration related to ongoing disenfranchisement in multiple sectors of daily life (e.g., housing, employment, education) can place Black men, in particular, at greater risk for perpetrating IPV [23, 24]. Existing research argues that the elevated risk for violence is largely due to being historically, socially and economically disadvantaged, not increase propensity due to biological or cultural factors [18, 19, 23]. Altogether, there is a need to advance meaningful epidemiological research addressing the role structural inequalities play in the perpetration of IPV.

To date, very little empirical research has investigated relationships between structural racism and IPV perpetration, but several studies have examined its implications on community violence and injuries [25, 26]. For example, a study found that rates of firearm injury were highest among areas in Philadelphia that have experienced historical redlining, an indicator of structural racism in housing [25]. This study argued that racial segregation in unequal neighborhoods and processes of racialized housing availability may explain racial differences in violence perpetration [25]. Another study found that states with higher rates of Black homicide tended to have a greater exposure of structural racism [26]. Unnever, Stults, Messner [26] state that the unequal distribution of resources across racial groups is a racialized social force with increased risk of homicide for Black Americans. Leveraging this existing conceptual and empirical research, we argue that structural racism does not require the actions of individuals [20, 27, 28], but can foster harmful interpersonal and individual contexts (e.g., legitimizing racial discrimination), which can in turn, increase the risk of adverse outcomes [21], such as IPV perpetration.

## Emotional reactivity of discrimination and men’s use of violence

Structural racism fosters racial discrimination [20, 21], and there are several adverse health outcomes related to racial discrimination [29–32]; however, its relationship to IPV perpetration is burgeoning. Recent studies found that high levels of racial discrimination are associated with IPV perpetration among Black men [33–35] and Black couples [36–38]. Using the General Strain Theory [39] and Hostile Attribution Bias Theory [40], Sutton et al. (2020) found evidence that negative emotional reactions such as anger resulting from experiences of racial discrimination, and this “displaced anger” manifested as IPV perpetration. This study also suggested that racial discrimination may prime men to have hostile attribution bias, in which ambiguous behaviors from a romantic partner are perceived as hostile.

In addition to the General Strain Theory [39] and the Hostile Attribution Bias Theory [40], the Psychological Reactance Theory may provide another explanation for the relationship between racial discrimination and IPV perpetration. Specifically, the Psychological Reactance Theory suggests that “in response to a perceived threat, people will experience a motivational state characterized by distress, anxiety, resistance, and desire to restore that freedom.” [41, 42] Thus, men may perceive racial discrimination as threating and attempt to restore their freedom and masculinity by using aggression towards women. This evidence explaining relationships between racial discrimination and IPV perpetration are not intended to absolve perpetrators of the violence inflicted but instead offer additional insights into the broader social-structural context that shapes IPV perpetration to inform anti-racist approaches to IPV prevention.

## Research aims

Guided by the General Strain Theory [39], the Hostile Attribution Bias Theory [40], and the Psychological Reactance Theory [41, 42], this study aims to examine associations of structural racism and IPV perpetration and the mediating effect of racial discrimination, stress, and optimism among Black and white men transitioning into parenthood. We hypothesized that, at baseline, Black men would experience more structural racism than white men. We also hypothesized that structural racism would be associated with more experiences of racial discrimination, higher severity of stress, and lower optimism at baseline. We also hypothesized that structural racism at baseline would be associated with IPV perpetration at the 6- and 12-month follow-up period. We also hypothesized that more experiences of racial discrimination at baseline would be associated with a higher severity of stress and lower optimism at baseline, which would be associated with IPV perpetration at the 6- and 12-month follow-up period. Finally, we hypothesized that the relationship between structural racism at baseline and IPV perpetration at the at the 6- and 12-month follow-up period would be partially mediated through racial discrimination, stress, and optimism.

## Methods

The current study linked census tract data with individual-level data from a prospective cohort of pregnant adolescents and their male partners [43]. From 2007 to 2011, 296 female-male couples (*N* = 592 participants) were recruited from women’s healthcare clinics in Connecticut (e.g., OB/Gyn, pediatric clinics). Couples were eligible if: (a) the female was in the second or third trimester at baseline, (b) both partners reported being in a romantic relationship with each other; (c) the female aged 14–21 years old at baseline; male at least 14 years at baseline; (d) both reported being the biological parents of the unborn baby; (e) both agreed to participate, and (f) both were able to speak English or Spanish.

Participants were informed about the study at women’s healthcare clinics. If only one participant was informed, they were asked to provide information to their partner from the research assistant (i.e., majority of couples were recruited by first recruiting the female participant and having her refer her male partner). If eligible for the study, participants were scheduled for a baseline interview. At baseline, written informed consent for all participants was obtained and parental consent was not obtained since CT regulation allows pregnant adolescents to be medically autonomous. Participants completed computer-assisted interviews in a private research office and were remunerated $25 for each time point (i.e., pregnancy; 6-months postpartum; and 12-months postpartum). All procedures were approved by the [masked for peer review] Institutional Review Board.

While dyadic or couple-level data were collected from pregnancy to 12-months postpartum, the current study only analyzed longitudinal data among the male partners to understand IPV perpetration. In this study, we sought to examine the implications of structural racism on Black men’s IPV perpetration with white men’s experiences as a comparison group.

## Measures

**Structural Racism.** Structural racism was assessed using a latent variable approach with four observed indicators at the city-level: (1) racial residential segregation, (2) education inequity, (3) income inequity, and (4) employment inequity. Latent variable indicators were based on Chantarat, Van Riper, Hardeman [44] research demonstrating multiple dimensions through which structural racism operates. Each of the observed indicators were calculated based on 5-year estimates from the American Community Survey from 2007 to 2011. The Black-white residential segregation was measured with the index of dissimilarity [44]. Education equity was measured by -white ratio of individuals aged 25 years and over who have completed a Bachelor’s degree or higher [44]. Income equity was measured by the index of concentration at the extremes [44]. Employment equity was measured by the Black-white ratio of civilians in the labor force aged 25–64 years. These four indicators were linked to individual-level data based on the participant’s census tract. Cities and census tracts were identified by using the geocodes from their residential address.

**Racial Discrimination.** Racial discrimination was assessed at the baseline assessment using 18-item Daily Life Experiences Scale [45]. This scale assesses whether participants experienced race-based experiences of unfair treatment in the past year [45]. Participants were asked to rate their agreement with the statements on a scale from 0 = *Never* to 5 = *Once a week or more*. Responses were summed to form a total score with a Cronbach’s alpha = 0.93.

**Stress.** Stress was assessed at the baseline assessment using the 10-item Perceived Stress Scale (PSS) [46]. This scale measured the frequency of stress and anxiety within the past month. Participants were asked to rate the stress frequency on a scale ranging 0 = *Never* to 4 = *Very Often.* Responses were summed to form a total score with a Cronbach’s alpha = 0.73.

**Optimism.** Optimism was assessed at the baseline assessment using 4-item Life Orientation Scale-Revised (LOT-R) [47]. Participants were asked statements about their outlook and expectations in life and responses were measured using a 5-point Likert scale, ranging from 1 = *Disagree a lot* to 5 = *Agree a lot*. Responses were summed to form a total score with a Cronbach’s alpha = 0.80.

**Emotional IPV Perpetration.** Emotional IPV perpetration was assessed at the 6-and 12-follow-ups using emotional or psychological IPV items (i.e., swear at you, call you names, or insult you) from the revised Conflict Tactics Scale-2 short form [48]. Participants were asked if they used the behavior towards their current partner (i.e., perpetration). Response options were 1 = Yes and 0 = No.

**Socio-demographics.** Socio-demographics were self-reported by participants: age (i.e., continuous score in years), race and ethnicity (i.e., Non-Hispanic Black, Non-Hispanic white), employment (i.e., Employed Part-Time, Employed Full-Time, Not Working), and highest level of education completed (i.e., Less than High School, High School or GED Completed, Some College, College Completed).

### Data analysis

Descriptive statistics (e.g., frequency, means [standard deviations]) were calculated to describe characteristics of the sample. Pearson correlation coefficients were used to assess relationships between racial discrimination, stress, optimism, and IPV perpetration. Descriptive analyses were conducted using SPSS v29. Maps were created using ArcGIS.

Structural racism was operationalized as a latent variable composed of four observed indicators (i.e., racial residential segregation, education inequity, income inequity, and employment inequity). Since individuals are nested within the geographic units where the structural racism measures were collection such as cities. Therefore, Black male participants in the same city have the same value for their latent indicator. We accounted for this non-independence and clustering while establishing the latent variable. Standard goodness of fit statistics for the confirmatory factor analysis was used to determine the model’s fit: (1) factor loadings greater than 0.40; (2) a RMSEA less than 0.08; and (3) a CFI at least 0.90 [49]. 

Structural equation models were conducted to examine structural racism as a predictor of racial discrimination, stress, optimism, and emotional IPV perpetration while accounting for individual-level covariates. The structural equation model predicting IPV perpetration were evaluated for goodness of fit based on: (1) a RMSEA less than 0.08; (2) a CFI at least 0.90, and a (3) TFI at least 0.90 [49]. Since IPV perpetration was a dichotomous variable, logistic regression was used. Individual-level socio-demographics were included as covariates in the regression models. Using the Monte Carlo method, the indirect effect was estimated for the full model. Regressions were conducted using Mplus v8.2.

## Results

Table 1 displays demographic characteristics of 175 men who identified as Non-Hispanic Black or white. The racial and ethnic makeup of the sample was Black or African American (82%) and White (18%). The employment status for the sample was unemployed (41.71%), part time (28.57%), and full time (28.57%). The average age was 21.3 years (SD = 4.05 years).

**Table 1:** Socio-demographics of men transitioning to parenthood

Variables	Overall (*N* = 175)	Race and ethnicity, and gender		*p*-value
		Black Men (*n* = 144)	White Men (*n* = 31)	
**Age**, *M (SD)*, years	21.3 (4.05)	21.44 (4.13)	23.06 (4.97)	0.06
**Education**, *M (SD)*, years	11.84 (1.89)	12.05 (1.88)	12.1 (1.88)	0.90
**Employment**, *N (%)*				0.01
Working full-time	50 (28.57)	34 (68.0)	16 (32.0)	
Working part-time	50 (28.57)	41 (82.0)	9 (18.0)	
Not Working	73 (41.71)	68 (93.15)	5 (6.8)	
**Racial discrimination**, *M (SD)*	0.29 (0.70)	0.34 (0.75)	0.11 (0.31)	0.01
**Stress**, *M (SD)*	15.39 (6.57)	15.60 (6.56)	14.45 (6.62)	0.37
**Optimism**, *M (SD)*	11.32 (2.33)	11.48 (2.33)	10.61 (2.23)	0.05
**IPV perpetration**, *N (%)*				0.57
Yes	47 (32.41)	41 (87.23)	6 (12.77)	
No	98 (67.58)	82 (83.67)	16 (16.32)	

^a^Row percentages shown

^b^*p*-values are results from t- or Chi-square tests

**Fig. 1:**
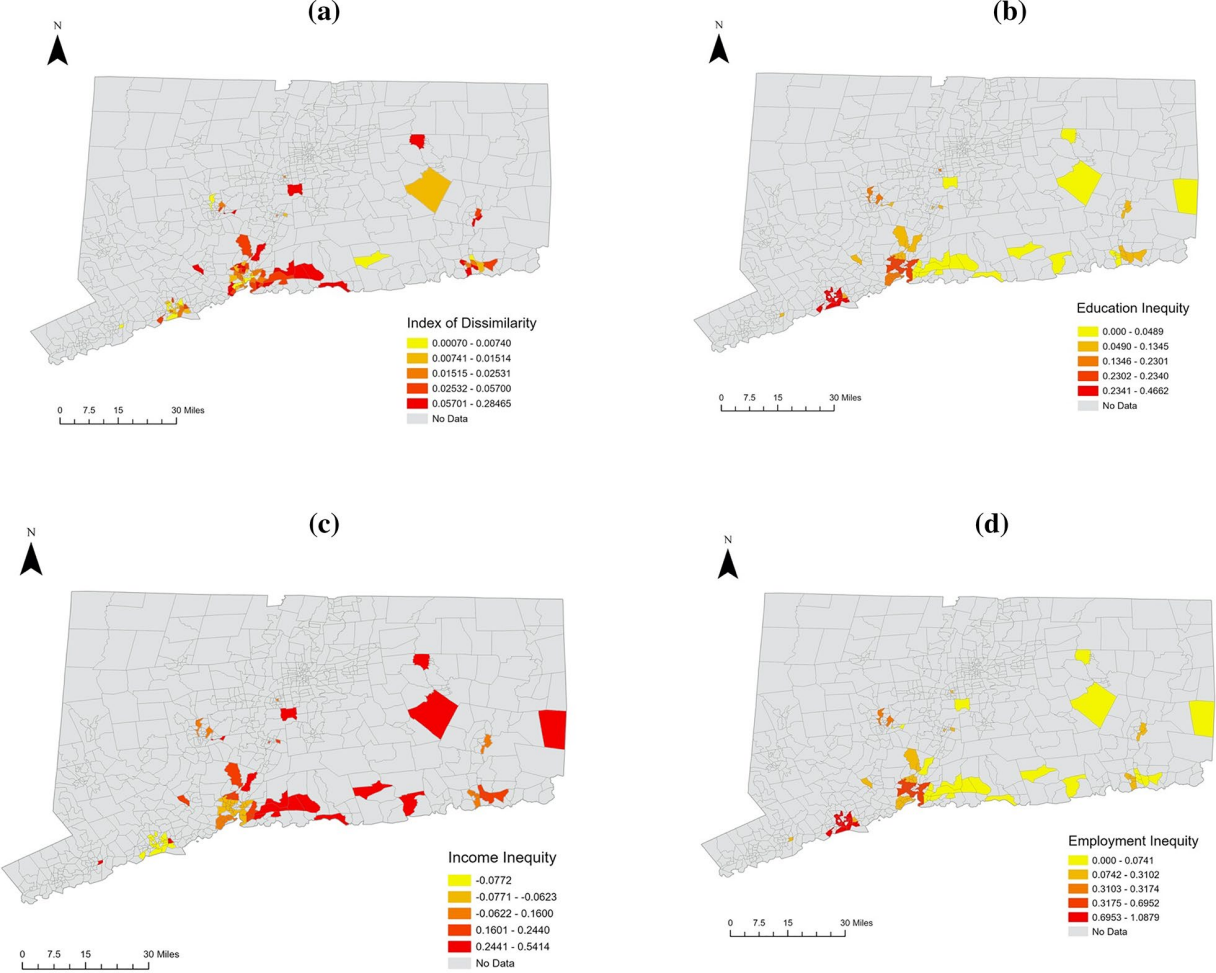
** a**-**d** Spatial distribution of structural racism latent indicators (i.e., income equity, education equity, employment equity, and index of dissimilarity)

Figure 1a-d depict the spatial distribution of each structural racism indicator. There were 24 cities comprising 104 total census blocks. The average score and range for each indicator was: (1) racial residential segregation [M (*R*) = 0.04 (0, 0.28); (2) education inequity [M (*R*) = 0.22 (0, 0.47), (3) employment inequity [M (*R*) = 0.52 (0, 1.09), and (4) income inequity [M (*R*) = 0.08 (−0.08, 0.54).

Table 2 demonstrates correlations among study variables. Racial discrimination was positively correlated with stress (*r* =.13, *p* <.05). Stress was also negatively correlated with optimism (*r*=-.25, *p* <.01) and positively correlated with 6-and 12-month follow-up IPV perpetration (*r* =.22, *p* <.01).

**Table 2:**
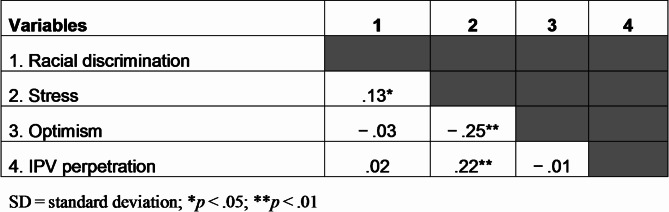
Correlations with discrimination, stress, optimism, and emotional IPV perpetration

## Assessing fit for measurement and structural models

The measurement model for the structural racism latent variable was assessed to confirm that the observed variables loaded adequately. The overall fit of the measurement model was satisfactory, CFI = 0.99, TLI = 0.99, and RMSEA = 0.02, with factor loadings greater than 0.40.

**Fig. 2:**
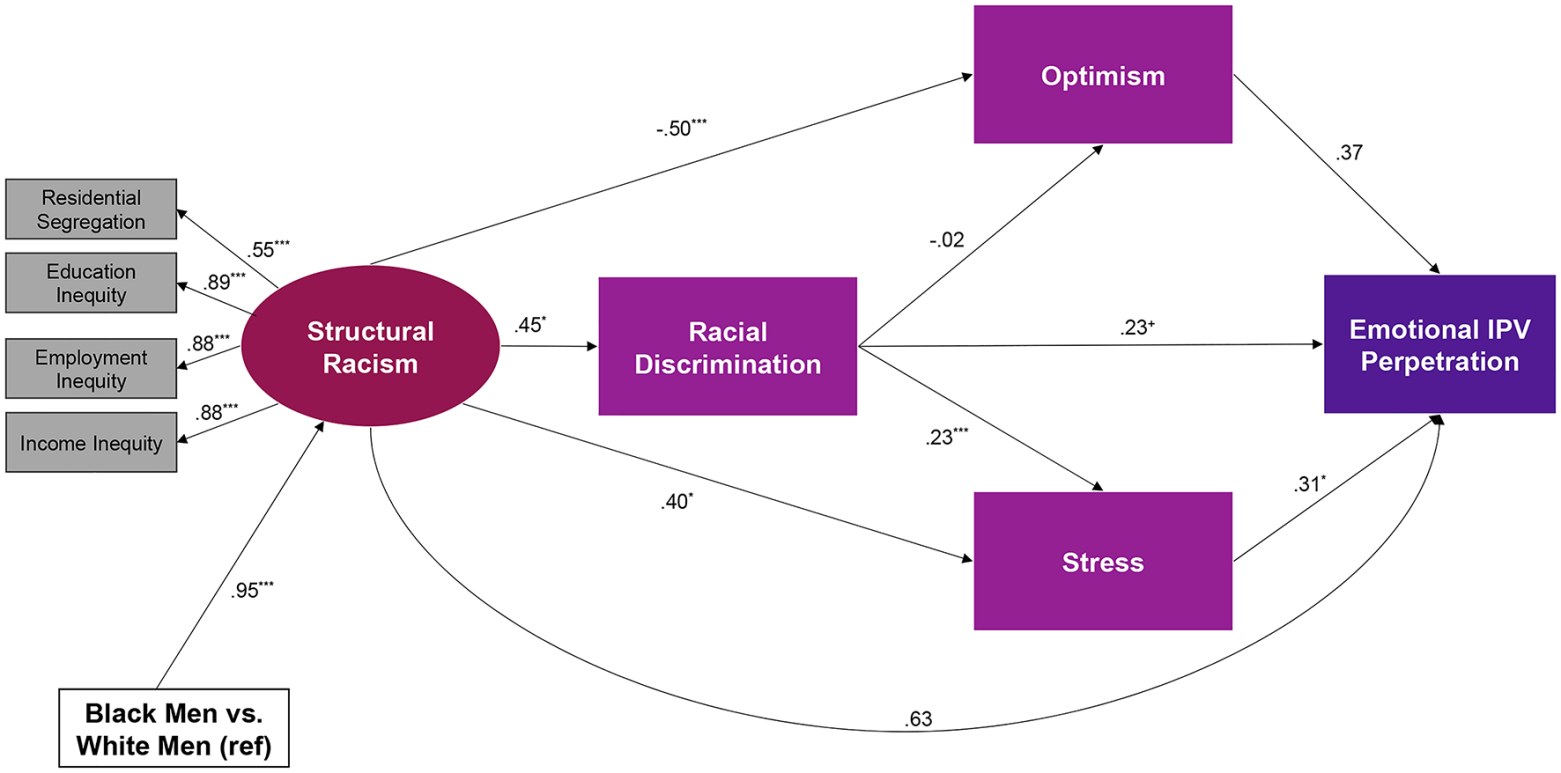
Path model displaying direct paths of structural racism, racial discrimination, stress, optimism, and emotional IPV perpetration among 175 Black and white men. Standardized estimates shown. ^+^*p* =.06; *p <.05; **p <.01; ***p <.001

Next, the overall fit for the structural model was assessed for 6-and 12-month follow-up IPV perpetration (Fig. 2) controlling for individual-level covariates. A satisfactory fit was found CFI = 0.92, TLI = 0.93, RMSEA = 0.07. This model accounted for 15% of the variance in IPV perpetration.

### Regression associations of structural racism to IPV perpetration

Figure 2 presents direct and indirect paths of structural racism with discrimination, stress, optimism, and 6-and 12-month follow-up IPV perpetration.

Six direct paths were statistically significant. Compared to white men, Black men were more likely to experience structural racism (B = 0.95, *p* <.001). Structural racism was associated with more racial discrimination (B = 0.45, *p* <.05), more stress (B = 0.40, *p* <.05), and less optimism (B=−0.50, *p* <.001). Racial discrimination was associated with more stress (B = 0.23, *p* <.05) and marginally associated with a greater likelihood to perpetrate emotional violence against a female partner at the 6-and 12-month follow-up period (B = 0.23, *p* =.06). Stress was associated with a greater likelihood to perpetrate emotional violence against a female partner at the 6-and 12-month follow-up period (B = 0.31, *p* =.05).

The indirect path from structural racism to 6-and 12-month follow-up IPV perpetration via racial discrimination and stress was marginally significant (B = 0.05, *p* =.07).

There were three non-statistically significant direct paths. Racial discrimination did not relate to optimism (B=−0.02, *p* =.23). Structural racism (B = 0.63, *p* =.65) and optimism (B = 0.37, *p* =.56) did not directly relate to emotional IPV perpetration at the 6-and 12-month follow-up period.

## Discussion

This is one of the first studies to examine the effects of structural racism on emotional IPV perpetration among U.S. Non-Hispanic Black and white men transitioning into parenthood. In our study, we found that structural racism, conceptualized as a latent variable comprising racial residential segregation, education inequity, income inequity, and employment inequity, was associated with more racial discrimination and stress, and lower optimism. We also found that the relationship between structural racism and emotional IPV perpetration was partially mediated through racial discrimination and stress. While the sample size prohibited multigroup structural equation modeling techniques by race and ethnicity, structural racism was positively associated with being a Non-Hispanic Black man compared to being a Non-Hispanic white man in this sample. Altogether our complex results can inform future public health efforts adopting an anti-racist approach to the prevention of IPV perpetration among U.S. men.

Our meditating relationship linking structural racism to IPV perpetration through racial discrimination and stress aligns with previous research on the Psychological Reactance Theory [41]. According to this theory, experiencing the detrimental effects of structural racism in daily life (e.g., education, employment) can create an interpersonal context where Black men are facing racial discrimination and oppression. By perceiving racial discrimination as a threat, Black men become stressed and use aggression against a female partner to restore their freedom and masculinity. The ability to restore one’s masculinity might be poignant for Black men who often experience marginalized masculinity [50]. While there can be significant variation in how masculinity is performed among Black men [51], hegemonic masculinity, a prevalent theoretical explanation for IPV perpetration [52–54], and most illustrated by being a primary material provider, physical dexterity, and sexual prowess [53, 54], is difficult to obtain by Black men due to the historical legacy of structural racism [23, 55]. Given that the effects of structural racism are often times outside of their control, an upstream public health intervention to address men’s use of IPV would be to dismantle structural racism in policies and services by reducing unequal distribution of resources in education, employment, housing, and income. For example, greater investment in funding to expand the supply and viability of rental housing, increase earned income tax credit, equitable raises for low-wage work, and equitable workforce development programs [56], have the potential to address structural racism and indirectly reduce IPV perpetration.

It is worth noting the paths from structural racism to IPV perpetration through racial discrimination were statistically significant, and the General Strain Theory [39] may provide an explanation these aforementioned relationships. According to the General Strain Theory [39], stressful life events and stressors like racial discrimination can produce negative emotions. As a result, men may engage in violence to cope with these negative feelings. Though negative emotions were not explicitly included in our model, it is possible that anger resulting from experiences of racial discrimination and structural racism could have manifested as emotional IPV against a female partner. This potential explanation aligns with prior research demonstrating that anger mediated the relationship between racial discrimination and physical IPV perpetration among Black men [33]. Overall, extensive research indicates that batterer intervention programs utilizing traditional Eurocentric paradigms are ineffective at reducing IPV perpetration [18], as such, our findings offer a potential modification for future IPV prevention programs which requires taking anti-racist stand to violence prevention. For example, batterer intervention programs may benefit from discussing the historical legacy of structural racism with Black men who use violence, and also address more positive coping mechanisms to discrimination and stress.

The study’s limitations need to be considered during research interpretation and dissemination. The age range of the sample included adolescents and young adults; thus our findings may have limited generalizability to older adults. The current study examined only emotional IPV perpetration and no other subtypes of IPV (e.g., physical, sexual) nor the chronicity of IPV perpetration. Therefore, our findings can only have implications for the prevention of emotional IPV perpetration. The data collection for IPV perpetration was self-reported, thus it is subject to underreporting due to the social desirability bias. Further, we used the short form of the CTS2 because IPV was one of a large battery of measures asked and we wanted to minimize participant burden. When compared to the full psychological aggression scale, the short form version performs adequately with a *r*=.77 [57]. Further, we have used this short form in several other studies examining the role of Psych IPV on health outcomes, and found expected relationships demonstrating predictive validity [58, 59]. Use of longitudinal data to analyze relationships of structural racism and IPV perpetration is a considerable study strength, however, we did not examine the impact of structural racism on trajectories of IPV perpetration among men. Prior research suggests that IPV perpetration can vary across time and context, thus future research should examine whether structural racism contributes to changes in IPV perpetration. Prior research has primarily used states and counties as the statistical areas for structural racism, however the current study used census block group and city-level data. While each of these different statistical areas can capture the heterogeneity of structural racism, smaller areas such as census tract and public use microdata areas might be able to capture the diversity of experiences in structural racism better [44]. The current study used previously validated measures for a structural racism latent variable, however the current variable does not capture every potential dimension of structural racism (e.g., healthcare, voting). Due to sample size constraints, the current study focused on Black and white men, but future studies should explore these relationships with additional ethnoracial groups (e.g., Hispanic men). While the legacy of structural racism has an ever-present in today’s society, our individual-level data was collected between 2007 and 2011, thus it is possible that patterns of emotional IPV perpetration might have changed since this data was originally collected.

## Conclusions

This study provides evidence of the ways in which structural racism in housing, education, income, and employment can contribute to men’s use of aggression and violence against a female partner. While the sample size limited our ability to investigate multigroup structural equation modeling, our model showed that Non-Hispanic Black men experienced more structural racism than Non-Hispanic white men, which related to men’s use of aggression by navigating their stress that stemmed from their experiences of racial discrimination. If our findings are replicated in studies with larger sample sizes, it will provide substantial support for future IPV prevention programs to adopt an anti-racist approach by working with communities and systems to dismantle structural racism.

## Data Availability

Some data is publicly available and can be extracted from the American Community Survey. However, some data cannot be shared to protect the privacy of participants.
